# Apoptosis and apoptotic mimicry in *Leishmania*: an evolutionary perspective

**DOI:** 10.3389/fcimb.2012.00096

**Published:** 2012-07-12

**Authors:** Charbel N. El-Hani, Valéria M. Borges, João L. M. Wanderley, Marcello A. Barcinski

**Affiliations:** ^1^Laboratório de Ensino, História e Filosofia de Biologia, Instituto de Biologia, Universidade Federal da BahiaSalvador, Brazil; ^2^Centro de Pesquisa Gonçalo Moniz, Fundação Oswaldo CruzSalvador, Brazil; ^3^Faculdade de Medicina, Universidade Federal da BahiaSalvador, Brazil; ^4^Instituto Nacional de Ciência e Tecnologia de Investigação em ImunologiaSalvador, Brazil; ^5^Pólo Universitário Macaé, Universidade Federal do Rio de JaneiroMacaé, Brazil; ^6^Laboratório de Biologia Celular, Instituto Oswaldo CruzRio de Janeiro, Brazil

**Keywords:** *Leishmania* spp., apoptosis, apoptotic mimicry, evolution, altruism, unicellular parasites

## Abstract

Apoptotic death and apoptotic mimicry are defined respectively as a non-accidental death and as the mimicking of an apoptotic-cell phenotype, usually by phosphatidylserine (PS) exposure. In the case of the murine infection by *Leishmania* spp, apoptotic death has been described in promastigotes and apoptotic mimicry in amastigotes. In both situations they are important events of the experimental murine infection by this parasite. In the present review we discuss what features we need to consider if we want to establish if a behavior shown by *Leishmania* is altruistic or not: does the behavior increases the fitness of organisms other than the one showing it? Does this behavior have a cost for the actor? If we manage to show that a given behavior is costly for the actor and beneficial for the recipient of the action, we will be able to establish it as altruistic. From this perspective, we can argue that apoptotic-like death and apoptotic mimicry are both altruistic with the latter representing a weaker altruistic behavior than the former.

## Recognition of microbial pathogens and apoptotic cells

Microbial pathogens and apoptotic cells are efficiently cleared by phagocytes. In both situations, however, the phagocytic process is distinguished by one crucial difference: while the former activates a host protective inflammatory response, the latter induces a non-inflammatory response (Torchinsky et al., 2010). The inflammatory response is mediated by receptors expressed by cells of the innate immune system, collectively known as Pathogen Recognition Receptors (PRR), which recognize ligands present on the surface of microorganisms known as Pathogen Associated Molecular Patterns [(PAMPs) (Kawai and Akira, 2009)]. On the other hand, apoptotic cells are engaged in phagocytosis via “find-me” and “eat-me” signals, recognized by different receptors present on professional and non-professional phagocytes (Fadok et al., 2001; Hoffmann et al., 2001; Ravichandran, 2010). The engulfment of apoptotic cells is followed by the release of anti-inflammatory cytokines by the phagocyte (Fadok et al., 1998b; Huynh et al., 2002). In 1989 Charles Janeway (1989) proposed the existence of the PRRs/PAMPs recognition system and the idea of self and infective non-self discrimination. In less than 10 years after Janeway's proposal, his hypothesis was supported by evidence showing an exquisite susceptibility to fungal infection in *Drosophila melanogaster* carrying a mutation in a so called “Toll” receptor (Lemaitre et al., 1996) and confirmed by the cloning and functional characterization of a human homologue of “Toll”, now defined as Toll-like receptor-4 (TLR-4) (Medzhitov et al., 1997). The system of PRRs, as now described, is composed of a large family of membrane-bound TLRs located on the cell surface or on intracellular vesicles as well as of cytosolic molecules such as retinoic acid-inducible gene-I (RIG-I)-like receptors (RLRs) and nucleotide-binding oligomerization domain (NOD)-like receptors (NLRs) that recognize intracellular PAMPs. Activation of the above described PRRs by PAMPs results in an inflammatory response due to the expression of several immune and inflammatory genes following activation of NF-κB or mitogen-activated protein kinases (MAPKs) (Akira et al., 2006). The process of endocytosis of apoptotic cells activates different pathways and depends on “find-me” and “eat-me” signals, respectively released and exposed by the apoptotic cells. “Find-me” signals are chemoattractants which induce migration of phagocytes to the proximity of dying cells while “eat-me” signals, when recognized by different receptors, serve as ligands for the rapid and efficient internalization of apoptotic cells or corpses and the induction of synthesis and release of anti-inflammatory cytokines (Fadok et al., 1998a; Ravichandran, 2010; Gregory and Pound, 2011). Of the several described “eat-me” signals, undoubtedly phosphatidylserine (PS) on the outer leaflet of the plasma membrane is the most important one (Fadok et al., 1992; Hoffmann et al., 2001). Exposure of PS is also the one that best characterizes a cell as being in the process of apoptotic death (Fadok et al., 1998a) and since it is also the one that defines apoptotic mimicry, it deserves some additional considerations. Exposed PS plays a role in the recognition and engulfment phases of the phagocytic process. In the last few years the search for one specific receptor for exposed PS lost momentum (Fadok et al., 2000; Henson et al., 2001) and now a days it is almost consensually accepted that several different molecular entities, in professional and non-professional phagocytes, are able to recognize PS directly or opsonized by soluble factors (Bratton and Henson, 2008). BAI1, TIM-4 and stabilin-2 have been described as PS receptors (Miyanishi et al., 2007; Park et al., 2007, 2008) while MFG-E8 (lactadherin) which binds to αv integrins (Hanayama et al., 2002) and Gas-6 which binds tyrosine kinase receptors of the Mer family on the phagocyte surface (Ishimoto et al., 2000) and β2-GPI which binds to a still unknown receptor (Balasubramanian and Schroit, 1998) are the best characterized soluble factors bridging apoptotic cells and phagocytes via PS. The dynamics of apoptotic cell recognition and engulfment includes loss of “don't-eat-me” signals followed by the appearance, in addition to exposed PS, of a functional phagocytic synapse with different tethering receptors, including PRRs such as CD-14 (Hoffmann et al., 2001; Brown et al., 2002; Devitt et al., 2003). Signaling through PS receptors leads to activation of the small GTPase Rac followed by modifications of the phagocyte cytoskeleton, formation of the membrane cup and subsequent target internalization. Studies in *C.elegans, D. melanogaster* and mammalian cells allowed the description of two key evolutionary conserved Rac-dependent pathways for apoptotic cell engulfment (Reddien and Horvitz, 2004; Elliott and Ravichandran, 2010; Kinchen and Ravichandran, 2010).

Independently of its role in phagocytosis, PS also signals for an anti-inflammatory response by the organism in which apoptotic cell death is occurring. This effect is due to the suppression of pro-inflammatory mediators coupled to the induction of release of anti-inflammatory effectors such as TGF-β, IL-10 and PGE-2 (Fadok et al., 1998b; Huynh et al., 2002).

## Apoptotic death in unicellular organisms

During the mid-nineties, three different groups described apoptotic-like death in three different species of trypanosomatids: *Trypanosoma cruzi* (Ameisen et al., 1995), *Trypanosoma brucei* (Welburn et al., 1996) and *Leishmania amazonensis* (Moreira et al., 1996), etiological agents of important endemic neglected diseases, respectively Chagas' Disease, African *trypanosomiasis* and *Leishmaniasis*. These papers were followed by a great number of descriptions of programmed cell death (PCD), apoptosis or apoptosis-like cell death in pathogenic and non-pathogenic unicellular organisms, occurring as part of the natural history of an infectious disease or resulting from environmental stresses to which the cells were exposed (Duszenko et al., 2006; Shaha, 2006; Deponte, 2008; Pollitt et al., 2010). Altogether these descriptions settled the fact that unicellular organisms can undergo an apoptosis-like program of cell death with some phenotypic features resembling PCD in multicellular organisms. Several important questions ranging from the biochemical machinery executing and controlling such events to the definition of selective pressures shaping the evolution of apoptosis-like death in unicellular organisms, remains to be better understood. However, the availability of tools for biochemically targeting the components of such type of death (Smirlis et al., 2010) and of bioinformatics-based comparisons between PCD pathways in different sample models of the phylogenetic evolutionary tree (Kaczanowski et al., 2011) is progressively diminishing this knowledge gap. An example is the definition of the molecular basis of DNA fragmentation observed in PCD in *Leishmania* spp. First described in *Leishmania donovani*, a pro-apoptotic endonuclease G (Endo G) displaying intrinsic nuclease activity migrates from the mitochondria to the nucleus when the cell is stimulated to undergo apoptotic death (Gannavaram et al., 2008). A homologous mitochondrial nuclease has been described to operate in *Leishmania infantum* apoptosis (Rico et al., 2009). Endoplasmic reticulum stress-induced apoptosis in *Leishmania major* is also dependent on the release of Endo G from mitochondria to nucleus via cytoplasm (Dolai et al., 2011). As a matter of fact, a comparative analysis of sequences of proteins functionally related to PCD, across a large number of unicellular eukaryotes, found PCD-related sequences in several unicellular lineages. The phylogenetic distribution of such sequences allowed the proposal that the PCD machinery operating in multicellular organisms has its origin in the early steps of eukaryote evolution (Nedelcu, 2009) and, as reviewed, death by apoptosis is phylogenetically conserved (Nguewa et al., 2004; Deponte, 2008). Different aspects of cell death in protozoan parasites were discussed in several recently published reviews (Luder et al., 2010; Smirlis et al., 2010; Van Zandbergen et al., 2010; Kaczanowski et al., 2011).

## Apoptotic mimicry and apoptotic death in *Leishmania* spp.

*Leishmania* spp. are trypanosomatids of special interest for the study of cell death mechanisms in pathogenic unicellular organisms: as amastigotes they multiply in acidic phagolysosomes generated by the internalization of the parasite in mammalian phagocytic cells, while as promastigotes they multiply and differentiate into infective forms in the gut of the sand fly vector. Thus, the demands of their digenetic life cycle imposes mechanisms for controlling cell growth and generating virulent forms in very different environments (El-Fadili et al., 2010). Furthermore, in order to complete their life-cycle and efficiently maintain disease progression and transmission, parasites must resist macrophage activation by the mammalian host innate and adaptive immune response as well as cope with the complex interactions with their sand fly vector in order to maximize transmission (Bates, 2008). Indeed, in the specific case of the murine model of infection with *Leishmania amazonensis*, both apoptotic mimicry and apoptotic-like death have been described and reviewed: the former in amastigotes and the latter in promastigotes (De Freitas Balanco et al., 2001; Wanderley et al., 2009; Wanderley and Barcinski, 2010). Apoptotic mimicry in amastigotes has been described as the capacity of these differentiated forms to expose PS without dying. As such, amastigotes engage exposed PS as an “eat-me” signal and are thus recognized and engulfed maintaining the anti-inflammatory properties of an apoptotic cell (Wanderley et al., 2006). The inhibition of host macrophage pro-inflammatory response and induction of anti-inflammatory cytokines generates an “easy-target” for parasite proliferation and disease progression. The receptors engaged and the signaling events following recognition of PS exposed on the parasite surface are still not defined.

Interestingly, amastigotes derived from lesions in Balb/c mice expose a higher density of PS moieties in their surface than amastigotes obtained from C57Bl/6 mice (Wanderley et al., 2006). This led the authors to investigate the immunological control of PS exposure and indeed it has been shown that amastigotes externalize PS and are rescued from death by a mechanism dependent on a non-polarized macrophage activation (simultaneous activation of iNOS and arginase I) induced by anti-*Leishmania* CD4^+^ T cells. The hypothesis is that the non-polarized response leads to PS externalization in response to NO induced stress and to parasite survival as a consequence of the simultaneous increase in polyamine synthesis. Indeed, host cellular immune response to infection with *Leishmania amazonensis* is usually very poor and does not follows the classical Th1/Th2 dichotomy described for infections with certain strains of *Leishmania maj*or (Heinzel et al., 1989). CD4^+^ T cells producing Th1 cytokines (IFNy and TNF), Th2 cytokines (IL-4, IL-5, and IL-13) as well as regulatory cytokines such as TGFβ and IL-10, has been described in mouse infected with *Leishmania amazonensis* (Afonso and Scott, 1993; Ji et al., 2002, 2003). Similar mechanisms have also been described to occur with other protozoan parasites such as *Toxoplasma gondii* (Seabra et al., 2004) and *Trypanosoma cruzi* (Damatta et al., 2007).

With promastigote forms the situation is different from the one described to occur with amastigotes. When progressing from the log to the stationary phase of *in vitro* culture, the frequency of PS-exposing promastigotes of *Leishmania major* increases. In addition, a sub-population of promastigotes collected from the midgut of the sandfly *Phlebotomus dubosqui* where shown to bind Annexin-V, being thus characterized as exposing PS. In both situations, PS-exposing parasites have suffered apoptotic death. Nevertheless, in an *in vivo* model of foot-pad swelling, the addition of apoptotic promastigotes to the infective inoculum of viable non-apoptotic parasites was shown to be a prerequisite for disease development. Apoptotic promastigotes were also capable of inactivating polymorphonuclear cells facilitating the intracellular survival of non-apoptotic infective parasites. Van Zandbergen et al. (2006) claim that the maintenance of viability of the non-apoptotic parasites by the apoptotic ones represents a form of altruistic interaction within the leishmanial population A similar situation has been described with promastigotes of *Leishmania amazonensis* (Wanderley et al., 2009). It has been shown that during normal *in vitro* metacyclogenesis and in the gut of *Lutzomya longipalpis*, a sub-population of metacyclic parasites dies by apoptosis. Apoptotic promastigotes (TUNEL^pos^) were only found in the anterior midgut to foregut boundary of infected sand fly vector, suggesting that this type of death does not occur at random but is rather part of the *in vivo* differentiation process. As part of the morphological changes occurring during apoptotic death, these parasites expose PS. PS-exposing, albeit non-viable, and the viable non-exposing parasites must be simultaneously added to the cell culture or inoculated in the mammalian host for an efficient *in vitro* macrophage infection or lesion progression in *in vivo* infections. In these situations, the viable non-exposing parasites are the infective forms, while the PS-exposing apoptotic sub-population inhibits macrophage inflammatory response. Here also our group considers this situation to be a “stable altruistic behavior in the context of a host-parasite interaction” (Wanderley et al., 2009).

## Apoptotic mimicry and apoptotic-like death as alternative forms of altruistic behavior

Considering that “[i]nterdisciplinary research on the social behavior of microorganisms is at the early stages of an exponential explosion” (West et al., 2006), issues concerning the application of social evolution theory to the understanding of cooperation and communication between unicellular eukaryotes and the selective advantages of cell death for a parasitic lifestyle are important matters to be focused. In spite of difficulties in the interaction between evolutionary biologists and microbiologists (West et al., 2006), social behavior of bacteria is an area in which progress is considerably greater than in parasitology. This unbalance is beginning to be overcome. An example is the interdisciplinary approach undertaken to outline “how an evolutionary framework can help make predictions about the ecological circumstances under which apoptosis [can] evolve” in a study with malaria parasites (Pollitt et al., 2010).

Altruistic apoptotic death [or apoptotic altruism without death (Barcinski et al., 2003)] and the eventual advantages and mechanisms employed for the usage of such a strategy in infections by unicellular pathogens have been proposed and discussed (Welburn et al., 1996; Debrabant and Nakhasi, 2003; Seed and Wenck, 2003; Duszenko et al., 2006; Pollitt et al., 2010). They have also been “a priori” disregarded by some due to a putative instability of such events in single celled organisms (James and Green, 2002). While altruism is conceived in everyday language as requiring altruistic motivations in a fundamental sense, in evolutionary biology an element of motive is not included in the definition of altruistic behaviors. The interpretation of parasite cell death as an altruistic behavior is not an anthropomorphic description. In fact, moral altruism as we discern in humans is a relatively recent arrival in the evolution of altruism, which can only evolve if altruistic behaviors exist in the first place and thus establish a selective regimen where motives can evolve as proximate psychological mechanisms bringing about altruistic behaviors (Sober and Wilson, 1998). We are certainly not proposing that psychological altruism can take place in *Leishmania*! Rather, our intention here is to apply multilevel selection theory, a key element in the construction of an expanded evolutionary synthesis (Wilson, 2010) in order to understand the evolution of the trait in terms of a conflict between within-group selection favoring selfish individuals and between-group selection favoring altruism. In this context, altruism is to be defined entirely in terms of fitness, with no appeal to motivation: an organism is said to behave altruistically when its behavior benefits other organisms, at a cost to itself. If we define altruism entirely in terms of survival and reproduction, measuring costs and benefits in terms of fitness, a behavior is altruistic if it increases the fitness of other organisms at the expense of the fitness of the actor. That is, when it behaves altruistically, an organism reduces the number of offspring likely to be produced by it, while boosting the number of offspring other organisms are likely to produce (Sober and Wilson, 1998; Okasha, 2009a,b). It is not hard to see why altruism seemed challenging for Darwinian evolutionary explanations, grounded on the survival of the fittest.

We can pinpoint what are the features we need to look at if we want to establish if a behavior shown by *Leishmania* is altruistic or not: does the behavior increases the fitness of organisms other than the one showing it? Does this behavior have a cost for the actor? If we manage to show that a given behavior is costly for the actor and beneficial for the recipient of the action, we will be able to establish it as altruistic. From this perspective, we can argue that apoptotic-like death and apoptotic mimicry are both altruistic with the latter representing a weaker altruistic behavior than the former.

Weak and strong altruism can be distinguished on the grounds of the difference between absolute and relative fitness (Wilson, 1977, 1990). Absolute fitness concerns the potential of survival and reproduction of individuals with a given genotype, while relative fitness relates to the potential for individuals with a given genotype to survive and reproduce as compared to the average fitness exhibited by the population containing them. Strong altruism reduces the absolute fitness of the acting organism or donor. When apoptotic-like death or cellular altruism takes place in *Leishmania*, the altruistic organism shows fitness equal to zero, and, thus, this is a strongly altruistic behavior.

However, a behavior needs not be extremely self-sacrificial to be regarded as altruistic. It can be weakly rather than strongly altruistic, only reducing the relative fitness of the donor. It can be even the case that the behavior boosts the absolute fitness of the donor, and, yet, is weakly altruistic, if it boosts the fitness of other organisms even more, thus reducing the donor's relative fitness (Okasha, 2009b). Moreover, if such a weak altruistic behavior appears in a population, it is likely to increase in frequency in relation to a strong altruistic behavior, since it is less detrimental to the individual organism than the latter.

Apoptotic mimicry can be interpreted as a weak altruistic behavior. However, we need to consider that all amastigotes of *Leishmania amazonensis* expose PS in their outer membrane, and, thus, it seems more correct to think that what we see now in these amastigote forms is the result of the fixation of a weak altruistic trait in the past. Assuming that the ancestral amastigote populations did not expose PS and, thus, PS-exposing variants appeared as novelties in those populations, and that it is likely that PS exposition has a price for the parasite (even though more research is needed on the topic), the amastigotes exposing PS would have its relative fitness reduced, no matter if they might still boost their own absolute fitness. Since the altruism in this case is of a weak form, it came to be fixed in the population, so that all *L.amazonensis* amastigotes now expose PS. Thus, the trait can be seen as a weak altruism fixed in the amastigote forms of *Leishmania amazonensis*. The fact that apoptotic-like death or cellular altruism has not been fixed in the promastigote forms, and we can still see sub-populations exposing and not exposing PS is, in turn, a consequence of the fact that this strong form of altruism cannot be fixed in the parasite, since promastigotes exposing PS have their fitness reduced to zero.

Finally, we should mention the interest that group selection models can show with regard to the explanation of the evolution of altruistic behaviors in *Leishmania*, given that they are detrimental to the individual organisms showing them. We need to consider, however, worries such as those expressed by West and colleagues (2006), namely, that to appeal to group selection might lead to confusion in the discussion about cooperation and social behaviors in general in microorganisms. We hold a different view from these authors in that the debates on group selection have been decisively solved in evolutionary biology during the 1960s–1980s. Rather, several relatively recent developments reinforced the debate in the last 15 years (Sober and Wilson, 1998; Nowak, 2006; Wilson and Wilson, 2007; Nowak et al., 2010). We cannot certainly say that group selection theory came to be widely accepted, but it is certainly present in the current scientific scenario as a theory worth debating and developing. While the developments mentioned above improved the theoretical plausibility of group selection as a significant evolutionary mechanism, we cannot neglect, also, the existence of empirical evidence supporting it, including evidence gathered in studies on microorganisms (Wade, 1976; Goodnight and Stevens, 1997; Swenson et al., 2000; Rainey and Rainey, 2003; Goodnight, 2005; Kerr et al., 2006). Also, contrary to West and colleagues (2006), we do not regard kin selection and group selection as mathematically identical. Group selection theory seems to be more general than kin selection (Sober and Wilson, 1998; Wilson and Wilson, 2007). If we assume that selection operates at several levels of the biological hierarchy, selection levels will often conflict: for instance, there is often tension between selection within groups and between groups. Since altruistic behaviors reduce individual absolute or relative fitness, if an altruistic behavior is to evolve, group selection should be sufficiently strong to counteract individual selection. In what conditions could this happen? First, the population should be divided into groups (in parasites, within hosts), i.e., there must be a population of groups. Second, the groups must vary in their proportion of organisms showing altruistic behaviors. Third, there should be a direct relationship between the proportion of altruists in a group and the fitness of the group, i.e., groups with more altruists should produce more individual offspring. Fourth, the progeny of the groups must mix or otherwise compete in the formation of new groups (Sober and Wilson, 1998).

How strong group selection is, relative to individual selection, depends on the amount of variation within and between groups. The most favorable scenario for the evolution of group-level adaptations, such as altruistic behaviors, is one of maximal variation between groups and decreased variation within groups. It is here that kinship enters the scene. If groups are formed by relatives, between-group variation is much higher than within-group variation, and, thus, kin selection can be seen as group selection taken to an extreme. The very proponent of kin selection (or inclusive fitness theory), William Hamilton, acknowledged that his original interpretation of it as an alternative to group selection was wrong (Hamilton, 1996). Moreover, group selection is more general than kin selection, since it can also take place between non-kin groups. Nevertheless, kin selection is indeed the kind of group selection more likely to take place in *Leishmania*, since intra-group (i.e., intra-host) variation in these parasites is not likely to be high, no matter if they show clonality or sexuality (Rougeron et al., 2010).

We should acknowledge that there is no experimental backing yet for the interpretation of apoptotic mimicry as a weak altruistic behavior. The main point in including this conjecture in the review is, precisely, to point out to research avenues that are opened up by this interpretation of the phenomenon and seems to be fruitful to pursue. For instance, it suggests that in order to elucidate the evolution of this trait it will be necessary to obtain more knowledge about the costs involved in PS exposition, so that we can provide an experimental backing to the interpretation of apoptotic mimicry as a weak altruistic trait. Moreover, we will need to know more about the regulation of this process, so that we can hypothesize about the origin of PS exposition as a variation in a previously non-exposing population. Moreover, if apoptotic death and apoptotic mimicry are not stochastic but regulated events with a functional role, we will need to investigate what are the mechanisms that govern the fate of one parasite which will die while the others will survive. These are important open questions for research on the evolution of apoptotic mimicry.

### Conflict of interest statement

The authors declare that the research was conducted in the absence of any commercial or financial relationships that could be construed as a potential conflict of interest.
